# Frequência escolar em indivíduos com paralisia cerebral no Brasil

**DOI:** 10.1111/dmcn.70341

**Published:** 2026-06-10

**Authors:** Ricardo Rodrigues de Sousa Junior, Luana Cristina da Silva, Ana Cristina Resende Camargos, Eliane Beatriz Cunha Policiano, Lívia Ferreira Coutinho Alonso, Nadia Badawi, Israt Jahan, Gulam Khandaker, Maria De Las Mercedes Ruiz Brunner, Paula Silva de Carvalho Chagas, Hércules Ribeiro Leite

**Affiliations:** ^1^ Programa de Pós‐Graduação em Ciências da Reabilitação, Escola de Educação Física, Fisioterapia e Terapia Ocupacional, Universidade Federal de Minas Gerais Belo Horizonte Brasil; ^2^ Registro Brasileiro Paralisia Cerebral‐ Grupo de Envolvimento do Público e do Paciente, Escola de Educação Física, Fisioterapia e Terapia Ocupacional, Universidade Federal de Minas Gerais Belo Horizonte Brasil; ^3^ Instituto de Pesquisa da Cerebral Palsy Alliance, Sydney Medical School, The University of Sydney Sydney New South Wales Austrália; ^4^ Escola de Saúde, Ciências Médicas e Aplicadas, Central Queensland University Rockhampton Queensland Austrália; ^5^ Central Queensland Hospital and Health Service Rockhampton Queensland Austrália; ^6^ Instituto Universitario de Ciencias Biomédicas de Córdoba (IUCBC), Centro de Investigación en Medicina Traslacional ‘Severo R. Amuchástegui’ (CIMETSA), Consejo Nacional de Investigaciones Científicas y Técnicas (CONICET) Córdoba Argentina; ^7^ Programa de Pós‐Graduação em Ciências da Reabilitação e Desempenho Físico‐Funcional, Faculdade de Fisioterapia, Universidade Federal de Juiz de Fora Juiz de Fora Brasil; ^8^ CanChild, McMaster University Hamilton Ontário Canadá

## Abstract

**Objetivo:**

Identificar fatores associados à frequência escolar entre crianças, adolescentes e jovens adultos brasileiros com paralisia cerebral (PC), utilizando dados populacionais do Registro Brasileiro de Paralisia Cerebral (RBPC).

**Método:**

Este estudo transversal analisou dados de 774 indivíduos com PC, com idades entre 4 e 21 anos, registrados no RPBC. As variáveis incluíram características sociodemográficas e clínicas (Sistema de Classificação da Função Motora Grossa, Sistema de Classificação das Habilidades Manuais e limitações de fala), além da frequência escolar. Foram realizadas análises descritivas e modelos de regressão logística multivariável foram aplicados para estimar *odds ratio* ajustadas (aORs) com seus respectivos intervalos de confiança de 95% (IC95%).

**Resultados:**

Entre os participantes, 76% frequentavam a escola, embora 62% não soubessem ler e escrever. Adolescentes com PC entre 13 e 21 anos apresentaram menor probabilidade de frequentar a escola em comparação àqueles entre 4 e 12 anos (aOR = 0,49; IC95% = 0,3–0,7), assim como aqueles classificados nos níveis IV e V do Sistema de Classificação da Função Motora Grossa em comparação aos níveis I a III (aOR = 0,29; IC95% = 0,1–0,4). Além disso, a presença de limitações de fala esteve associada à menor frequência escolar (aOR = 0,12; IC95% = 0,1–0,3).

**Interpretação:**

Embora as taxas de frequência escolar de indivíduos brasileiros com PC sejam relativamente altas em comparação a outros países de baixa e média renda, importantes barreiras persistem. O enfrentamento de aspectos relacionados ao suporte à comunicação e à inclusão relacionada à mobilidade é essencial para melhorar tanto a frequência escolar quanto o envolvimento significativo.

**Abreviações**
RBCP: Registro Brasileiro de Paralisia Cerebral
EPP: envolvimento do público e do paciente

**O que este artigo acrescenta**

- Setenta e seis por cento dos indivíduos com paralisia cerebral no Brasil frequentam a escola.
- A não frequência escolar está associada à maior idade dos participantes.
- A não frequência escolar está associada a limitações na função motora e na fala.

A participação pode ser definida como envolvimento e frequência em atividades da vida real.^1^ Quando as crianças se engajam em atividades prazerosas em diferentes contextos, sua saúde e bem‐estar melhoram.^2^ No entanto, no Brasil, um país de baixa e média renda, dados recentes têm demonstrado baixos níveis de participação entre crianças com deficiência, como aquelas com paralisia cerebral (PC), em diferentes contextos. Essa participação tende a diminuir com o aumento da idade e com maiores limitações de mobilidade.^3^ De acordo com a definição atualizada de PC por Dan et al.,^4^ essa população “… frequentemente apresenta comprometimentos primários e secundários em várias áreas do desenvolvimento e do funcionamento, o que pode impactar significativamente sua participação na vida diária …”.

A frequência escolar (aqui entendida como a presença física na escola) representa uma importante expansão do ambiente social da criança e favorece o desenvolvimento de habilidades acadêmicas e da vida cotidiana.^5^ Um dos objetivos de desenvolvimento sustentável das Nações Unidas é que, até 2030, “todas as meninas e meninos completem o ensino primário e secundário gratuito, equitativo e de qualidade, resultando em desfechos de aprendizagem relevantes e eficazes”.^5^ Crianças com PC podem enfrentar restrições na participação escolar, relacionadas à ausência de formação adequada dos professores, à falta de suporte educacional completo, a barreiras físicas e atitudinais, além de bullying e assédio.^6^ Apesar do estabelecimento de políticas educacionais voltadas à promoção da inclusão de crianças com deficiência em todos os contextos escolares, e da obrigatoriedade de cumprimento da Convenção das Nações Unidas sobre os Direitos da Criança e da Convenção sobre os Direitos das Pessoas com Deficiência, muitas barreiras à participação plena ainda persistem.^7^ Por exemplo, pessoas com deficiência apresentam menor frequência escolar, menores taxas de conclusão escolar, menos anos de escolaridade e níveis mais baixos de alfabetização.^6^ Esses desafios são ainda mais pronunciados em países de baixa e média renda.

Um estudo recente que apresentou dados sobre a frequência escolar de crianças com PC em Bangladesh revelou que, embora 1582 crianças estivessem em idade escolar, apenas 26,1% frequentavam a escola.^6^ Dados de outros países de baixa e média renda apresentaram resultados semelhantes quanto à frequência escolar de crianças com PC: no Sudão, 12,3%;^8^ no Benim, 23,3%;^9^ em Uganda, 30,0%;^10^ e na Índia, 40,0%.^11^ Adolescentes com PC, indivíduos com PC bilateral e aqueles com comprometimentos de fala apresentaram mais barreiras para frequentar a escola em Bangladesh.^6^ Crianças com PC com maior frequência de comorbidades, dificuldades de aprendizagem, comprometimento da acuidade visual e epilepsia apresentaram maior probabilidade de menor frequência escolar na Nigéria.^12^ A gravidade do comprometimento motor e das habilidades manuais também esteve associada a menores chances de frequência escolar.^6^, ^12^

No início do século, o Brasil não possuía um plano de sistema educacional voltado à inclusão de pessoas com deficiência, apesar da Constituição de 1988 garantir o acesso à educação sem discriminação.^13^ Esse cenário começou a mudar com o fortalecimento de movimentos em favor da inclusão impulsionados pela Declaração de Salamanca e pela Convenção da Guatemala.^14^ A Lei Brasileira de Inclusão da Pessoa com Deficiência, que assegura o direito de crianças com deficiência frequentarem todos os níveis de ensino, está completando 10 anos.^15^ Este foi um grande avanço para um país com 11 milhões de pessoas não alfabetizadas. Dos 151 milhões de indivíduos que frequentam a escola, 47,4% não concluíram o ensino médio.^16^ No Brasil, 22,3% das pessoas com deficiência não são alfabetizadas, um número quatro vezes maior do que na população brasileira com desenvolvimento típico.^16^ Dados sobre crianças com PC frequentando a escola no Brasil ainda são escassos. O estudo preliminar PartiCipa Brasil demonstrou que um em cada quatro indivíduos com PC em idade escolar estava fora da escola.^17^

Iniciativas para melhor compreender a epidemiologia, a inclusão e a participação de crianças com PC em países de baixa e média renda têm emergido, como o *Global Low‐ and Middle‐Income Countries Cerebral Palsy Registry*.^18^ Na América Latina, especificamente, o Registro Latino‐Americano de Paralisia Cerebral vem coletando dados de diversos países da região, sendo o Brasil o país mais populoso e com maior participação.^19^ O Registro Brasileiro de Paralisia Cerebral (RBPC) iniciou a coleta de dados em setembro de 2024.^20^ O objetivo deste estudo foi descrever a frequência escolar entre indivíduos com PC no Brasil e identificar fatores associados à frequência escolar. Essas informações são importantes para orientar políticas públicas e investimentos em na defesa dos direitos e inclusão escolar de indivíduos com PC no Brasil.

## MÉTODOS

Dados retrospectivos do RBPC foram utilizados neste estudo para descrever a frequência escolar de crianças, adolescentes e jovens adultos com PC no Brasil.

### Registro Brasileiro de Paralisia Cerebral

O RBPC é um sistema nacional de vigilância estabelecido em 2024 por meio de uma colaboração entre pesquisadores, profissionais, parceiros internacionais e um grupo ativo de envolvimento de pacientes e do público (EPP). Ele integra esforços globais, como o *Global Low‐ and Middle‐Income Countries Cerebral Palsy Registry* e o *Latin American Cerebral Palsy Register*, para ampliar o conhecimento sobre a epidemiologia da PC em países de baixa e média renda.^19^ O RBPC adota a Classificação Internacional de Funcionalidade, Incapacidade e Saúde como seu referencial organizador, permitindo uma compreensão mais abrangente do funcionamento humano ao incorporar atividades, participação, funções e estruturas do corpo, além de fatores pessoais e ambientais. O registro utiliza estratégias de coleta de dados tanto presenciais (por exemplo, hospitais, clínicas e centros de saúde) quanto remotas, em todas as cinco regiões do Brasil, em cidades urbanas e rurais. O RBPC registra indivíduos com PC de 0 a 100 anos de idade. O grupo EPP, incluindo adultos com PC, mães de crianças com PC, profissionais e pesquisadores, está envolvido desde a criação do registro, contribuindo para a seleção de variáveis e materiais de divulgação, garantindo que as perspectivas das pessoas com PC orientem as prioridades do projeto.^20^

### Participantes do estudo, critérios de inclusão/exclusão e coleta de dados

Os participantes do estudo foram indivíduos registrados no RBPC. Para este estudo, foram extraídos dados do RBPC de participantes com idades entre 4 e 21 anos. A idade escolar obrigatória no Brasil é de 4 a 17 anos. Neste estudo, ampliamos a faixa etária até 21 anos para também incluir aqueles matriculados em programas brasileiros de educação de jovens e adultos. Assim, entre agosto de 2024 e maio de 2025, 774 crianças e jovens foram incluídos no banco de dados do RBPC, provenientes de todas as cinco regiões do Brasil.

Informações detalhadas sobre o registro de indivíduos com diagnóstico confirmado de PC no RBPC foram publicadas previamente por nossa equipe.^20^ Os dados foram coletados por meio de entrevistas com o indivíduo com PC ou seus cuidadores principais (presencialmente em clínicas, centros de reabilitação, hospitais e centros de saúde, e de forma remota). Cada entrevista teve duração máxima de 30 minutos. Todos os pesquisadores receberam treinamento da equipe do RPBC para a coleta de dados. O formulário de coleta foi adaptado da versão recomendada pelo *Latin American Cerebral Palsy Register* e pelo *Global Low‐ and Middle‐Income Countries Cerebral Palsy Registry*, e foi aplicado por meio de uma plataforma online (REDCap, Vanderbilt University, Nashville, TN, EUA). Este formulário está disponível como Apêndice S1.

### Organização dos dados

Variáveis sociodemográficas e clínicas selecionadas foram extraídas do banco de dados do RBPC. As seguintes características sociodemográficas foram obtidas: idade e sexo do participante, região e estado do Brasil onde reside, frequência escolar (se o participante frequentava fisicamente a escola), nível de ensino (ensino primário, incluindo educação infantil, ensino fundamental I e II, geralmente denominado ensino fundamental no Brasil; e ensino secundário, geralmente denominado ensino médio), alfabetização (capacidade de ler e escrever), frequência em reabilitação, uso de dispositivos assistivos, informação sobre quem era o principal provedor financeiro da família, renda mensal domiciliar, nível educacional do principal responsável ou cuidador e acesso aos serviços de saúde público e privado. Essas informações foram obtidas de acordo com as respostas fornecidas pelos pais ou cuidadores durante as entrevistas do RBPC.

Foram coletadas características clínicas, como nível de mobilidade (Sistema de Classificação da Função Motora Grossa‐ *Gross Motor Function Classification System* [GMFCS]), nível de habilidade manual (Sistema de Classificação da Função Manual‐ *Manual Ability Classification System* [MACS]), tipo motor da PC (espástica, discinética, atáxica, hipotônica ou mista), topografia da PC (unilateral ou bilateral) para PC espástica, comprometimentos associados (intelectual ou de fala) e presença de comorbidades (luxação de quadril, epilepsia, refluxo, problemas auditivos, deficiência visual e dificuldades de deglutição). Todas as características clínicas foram coletadas com base na percepção dos pais e cuidadores e confirmadas em prontuários médicos e por clínicos, quando disponíveis. Utilizamos a versão de relato familiar do GMFCS e o fluxograma de entrevista do MACS para coletar de forma acurada as percepções dos pais e cuidadores com base no desempenho dos participantes.^21^

### Análise estatística

Estatísticas descritivas (frequências ou médias com desvios padrão) foram inicialmente utilizadas para resumir as características demográficas, clínicas e contextuais dos participantes, incluindo idade, sexo, frequência em reabilitação, região e estado do Brasil, uso de dispositivos assistivos, alfabetização (isto é, a capacidade de ler e escrever), acesso aos serviços de saúde, tipo de PC, topografia da PC, nível do GMFCS e nível do MACS.

Para explorar as associações iniciais entre cada variável independente e a frequência escolar, foram realizadas análises bivariadas utilizando o teste do χ^2^. A partir da triagem bivariada, a frequência escolar (sim/não) foi definida a priori como variável dependente. Todas as variáveis sociodemográficas e clínicas consideradas como fatores candidatos foram baseadas em relevância clínica, plausibilidade teórica e evidências de estudos epidemiológicos prévios sobre PC. Essas variáveis incluíram: sexo (feminino/masculino); faixa etária (4–12 anos e 13–21 anos); nível de ensino (primário/secundário); alfabetização (sim/não); principal provedor financeiro (mãe ou pai/outros); renda domiciliar (abaixo vs acima de dois salários mínimos brasileiros: < R$3000/US$538); escolaridade dos pais ou cuidadores (ensino médio incompleto ou inferior vs ensino médio completo ou superior); tipo motor (espástico/outros); topografia (unilateral/bilateral); nível do GMFCS (deambuladores: níveis I–III; não deambuladores: níveis IV e V); nível do MACS (uso manual independente: níveis I e II; uso manual não independente, ou seja, necessitando de adaptações ou apoio do cuidador: níveis III–V); deficiência intelectual (sim/não); limitações visuais (sim/não); limitações de fala (sim/não); e comorbidades (nenhuma vs ≥1).

Modelos de regressão logística foram inicialmente ajustados para estimar associações brutas entre cada variável e a frequência escolar. Para evitar sobreajuste do modelo e garantir parcimônia, apenas variáveis que atenderam a dois critérios foram incluídas no modelo de regressão logística multivariável ajustado: (1) significância estatística nas análises bivariadas por χ^2^ (p < 0,05); e (2) justificativa conceitual com base na literatura prévia sobre participação escolar em crianças com PC.

Antes do ajuste do modelo, avaliamos a multicolinearidade entre todos os fatores candidatos utilizando o fator de inflação da variância. Todos os valores foram inferiores a 3, indicando ausência de multicolinearidade relevante.^22^, ^23^ No modelo ajustado, foram incluídas as variáveis estatisticamente significativas no modelo não ajustado. Os modelos finais foram apresentados como odds ratios (ORs) e odds ratios ajustadas (aOR) com intervalos de confiança de 95% (IC95%).

Os dados ausentes foram examinados quanto a padrões; adotou‐se uma abordagem de casos completos devido à baixa proporção de dados faltantes e à ausência de evidências de viés sistemático. Todas as análises foram realizadas utilizando o SPSS v26 (IBM Corp., Armonk, NY, EUA).

### Envolvimento do público e do paciente

O RBPC utiliza a metodologia de EPP. Esse grupo inclui quatro pesquisadores, seis mães de crianças com PC (uma no nível II do GMFCS; duas n nível III do GMFCS; três no nível V do GMFCS) e dois adultos com PC (nível IV do GMFCS). Seus papéis dentro do registro foram definidos com base na matriz de envolvimento do EPP.^24^ Para o presente estudo, duas mães do grupo principal foram convidadas. Seus papéis foram atuar como co‐pensadoras e parceiras na pesquisa.

O grupo EPP está envolvido desde a criação do RBPC e participou do processo de tradução e adaptação do formulário de coleta de dados, sugerindo a inclusão de perguntas relevantes e modificações nas opções de resposta de determinados itens. No presente estudo, os membros definiram a questão de pesquisa que orientou o estudo, com base na discussão das dificuldades dos pais em relação à participação escolar de seus filhos. Eles também auxiliaram na definição das variáveis a serem incluídas nas análises e contribuíram para a interpretação dos resultados e a disseminação dos achados por meio da elaboração de um infográfico.

### Considerações éticas

O estudo foi aprovado pelo comitê de ética da Universidade Federal de Minas Gerais (parecer n° 79576124.2.0000.5149). Todos os cuidadores dos indivíduos com PC ou os próprios indivíduos assinaram o termo de consentimento livre e esclarecido. Além disso, crianças e adolescentes capazes de compreender os objetivos do estudo também assinaram o termo de assentimento.

## RESULTADOS

### Características dos participantes do estudo

A média de idade dos participantes no momento do registro foi de 9 anos e 9 meses (DP = 4 anos e 3 meses), e 331 (42,8%) eram do sexo feminino. A Figura 1 apresenta a proporção de participantes em cada região e estado do Brasil. As principais características desse grupo estão apresentadas na Tabela 1.

**Figura 1:**
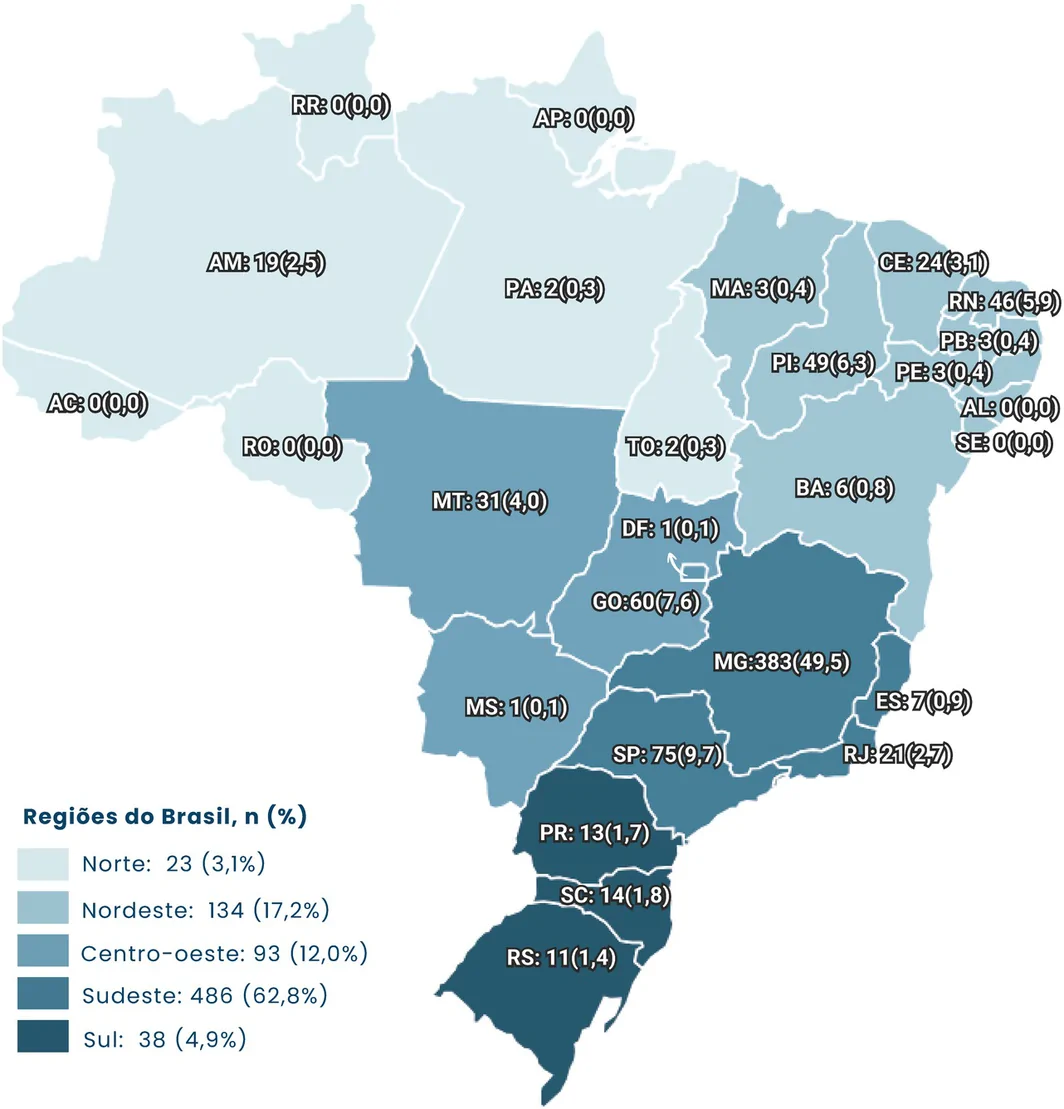
Proporção da amostra entre as regiões e estados brasileiros. AC, Acre; AL, Alagoas; AM, Amazonas; AP, Amapá; BA, Bahia; CE, Ceará; DF, Distrito Federal; ES, Espírito Santo; GO, Goiás; MA, Maranhão; MG, Minas Gerais; MS, Mato Grosso do Sul; MT, Mato Grosso; PA, Pará; PB, Paraíba; PE, Pernambuco; PI, Piauí; PR, Paraná; RJ, Rio de Janeiro; RN, Rio Grande do Norte; RO, Rondônia; RR, Roraima; RS, Rio Grande do Sul; SC, Santa Catarina; SE, Sergipe; SP, São Paulo; TO, Tocantins.

**Tabela 1:** Principais características dos participantes do RBPC incluídos no estudo (n = 774).

Características	*n* (%)	
Idade [mediana (intervalo interquartil	9 (7)	
Sexo		
Feminino	331 (42.8)	
Masculino	443 (57.2)	
Nível GMFCS		
I	74 (9.6)	
II	130 (16.8)	
III	112 (14.5)	
IV	149 (19.3)	
V	280 (36.2)	
Nível MACS		
I	102 (13.2)	
II	179 (23.1)	
III	107 (13.8)	
IV	129 (16.7)	
V	218 (28.2)	
Tipo de PC		
Espástica	592 (76.5)	
Atáxica	12 (1.6)	
Discinética	61 (7.9)	
Hipotônica	39 (5)	
Topografia da PC		
Unilateral	487 (62.9)	
Bilateral	90 (11.6)	
Acesso a serviços de saúde		
Público	630 (81.4)	
Privado	364 (47.0)	
Apoio da reabilitação		
Sim	743 (96)	
Não	53 (6.8)	
Apresenta todos os dispositivos de tecnologia assistiva		
Sim	391 (50.5)	
Não	170 (22.0)	
Parcialmente	182 (23.5)	
Alfabetização (idade)	**Sim**	**Não**
4–6	53 (29.9)	124 (70.1)
7–12	78 (27.6)	204 (72.4)
13–21	79 (33.4)	157 (66.6)
Total	210 (27.1)	485 (62.2)

*Note*: Dados ausentes: nível do GMFCS n = 29 (3,7%); nível do MACS n = 39 (5%); tipo de PC n = 70 (9%); topografia n = 197 (25%); acesso aos serviços de saúde n = 18 (2,3%); suporte de reabilitação n = 22 (2,8%); dispositivos de tecnologia assistiva n = 31 (4%); alfabetização n = 79 (10,7%).

Abreviações: RBPC, Registro Brasileiro de Paralisia Cerebral; GMFCS, Sistema de Classificação da Função Motora Grossa; MACS, Sistema de Classificação da Habilidade Manual.

Nesse grupo, 589 (76%) frequentavam a escola e 485 (62%) não eram alfabetizados; 74% desses indivíduos não alfabetizados tinham 6 anos ou mais (isto é, a idade esperada para alfabetização no Brasil). Entre todos os participantes, 567 (73,3%) apresentavam PC espástica, 429 (55,4%) foram classificados nos níveis III a V do GMFCS, 580 (74,9%) apresentavam comprometimentos de fala e 738 (95%) recebiam algum tipo de reabilitação no momento da participação na entrevista do RBPC. Mais detalhes são apresentados nas Tabelas 2 e 3.

**Tabela 2:** Fatores contextuais associados à frequência escolar entre crianças e jovens com paralisia cerebral no RBPC.

Fatores	Frequência escolar						
		Total (%)^a^	*n* (%)	Sim (%)	Significância do χ^2^	Odds ratio não ajustado (IC95%)^b^	*p*
Idade (anos)					0.002	Ref.	
4–12		575 (73.3)	98 (17.1)	447 (77.7)			
13–21		207 (26.7)	56 (27.1)	142 (68.6)		**0.57 (0.3–0.9)**	**0.03**
Sexo					0.57	Ref.	
Feminino		331 (42.8)	69 (20.8)	249 (75.2)			
Masculino		443 (57.2)	85 (19.2)	340 (76.7)		0.90 (0.3–1.2)	0.57
Nível escolar					0.17	Ref.	
Fundamental		435 (56)	40 (9.2)	395 (90.8)			
Médio		61 (7.9)	9 (14.8)	52 (85.2)		0.69 (0.4–1.0)	0.52
Alfabetização					0.001	Ref.	
Sim		210 (27.1)	15 (7.1)	195 (92.2)			
Não		485 (62.7)	125 (25.8)	358 (73.8)		0.58 (0.2–1.1)	0.16
Provedor primário					0.20	Ref.	
Mãe ou pai		574 (74.2)	104 (18.1)	447 (77.9)			
Outro		196 (25.3)	50 (25.5)	139 (70.9)		0.64 (0.4–1.9)	0.20
Nível de educação dos pais ou cuidadores					0.51		
Ensino médio incompleto ou abaixo		321 (41.5)	73 (22.7)	233 (72.6)		Ref.	
Ensino médio complete ou ensino superior		428 (55.3)	74 (17.3)	339 (79.2)		1.44 (0.9–2.2)	0.60
Renda (R$)					0.20	Ref.	
< R$3000		323 (41.7)	71 (22.0)	238 (73.7)			
> R$3000		412 (52.2)	77 (18.7)	327 (79.4)		1.26 (0.8–1.8)	0.20

Abreviações: RBPC, Registro Brasileiro de Paralisia Cerebral; IC, intervalo de confiança.

^a^ Dados ausentes: nível de ensino n = 278 (35%); alfabetização n = 79 (10,2%); principal provedor financeiro n = 4 (0,51%); renda familiar n = 39 (5,0%).

^b^ As variáveis incluídas no modelo final estão destacadas em negrito (isto é, aquelas significativas na análise bivariada.

### Fatores relacionados com a frequência escolar

#### Fatores sociodemográficos

Entre nossas variáveis independentes, idade e alfabetização foram as únicas variáveis sociodemográficas estatisticamente associadas à frequência escolar (p < 0,01) na análise bivariada preliminar (teste do χ^2^) (Tabela 2).

A frequência escolar foi maior em crianças de 4 a 12 anos em comparação com adolescentes e jovens adultos de 13 a 21 anos. Adolescentes e jovens adultos apresentaram *odds ratio* não ajustadas significativamente maiores de não frequentar a escola (OR não ajustada = 0,57 [0,3–0,9]; p = 0,01). A alfabetização não esteve significativamente associada à frequência escolar (p = 0,16). Esses resultados estão detalhados na Tabela 2.

#### Fatores clínicos

As características clínicas relacionadas à topografia da PC, nível do GMFCS, nível do MACS, presença de deficiência intelectual, limitações de fala e presença de outras comorbidades apresentaram diferenças estatisticamente significativas (p < 0,002) na frequência escolar na análise bivariada preliminar (Tabela 3).

**Tabela 3:** Fatores clínicos associados à frequência escolar entre crianças e jovens com paralisia cerebral no RBPC.

Fatores	Frequência escolar					
	Total (%)^a^	*n* (%)	Sim (%)	*Significância do χ* ^*2*^	Odds ratio não ajustado (IC95%)^b^	*p*
Tipo de PC				0.22	Ref.	
Espastica	567 (73.3)	16 (20.5)	447 (78.8)			
Outros tipos	105 (13.6)	88 (15.2)	116 (83.8)		1.40 (0.8–0.2)	0.22
Topografia				0.001	Ref.	
Bilateral	477 (62.9)	120 (24.6)	363 (74.5)			
Unilateral	90 (11.6)	2 (2.2)	88 (97.8)		4.5 (0.5–36.5)	0.15
Nível GMFCS				0.001	Ref.	
Deambulador (I–III)	316 (40.8)	26 (8.2)	287 (90.8)			
Não deambulador (IV‐V)	429 (55.4)	127 (29.6)	298 (69.5)		**0.36 (0.1–0.7)**	**0.01**
Nívle MACS				0.001	Ref.	
I‐II	282 (36.4)	20 (7.1)	260 (92.2)			
III–V	454 (58.7)	129 (28.4)	321 (70.7)		0.55 (0.2–1.7)	0.13
Deficiência intellectual				0.001	Ref.	
Não	199 (25.7)	23 (11.6)	176 (88.4)			
Sim	464 (59.9)	109 (23.5)	350 (75.4)		0.70 (0.3–1.3)	0.30
Limitações na fala				0.001	Ref.	
Não	158 (20.4)	4 (2.5)	153 (20.4)			
Sim	580 (74.9)	146 (25.2)	430 (74.1)		**0.20 (0.4–0.9)**	**0.03**
Comorbidades				0.001	Ref.	
Nenhuma	129 (16.7)	8 (6.2)	115 (16.7)			
Uma ou mais	627 (81)	146 (23.3)	474 (75.6)		0.55 (0.1–1.7)	0.30

Abreviações: RBPC, Registro Brasileiro de Paralisia Cerebral; IC, intervalo de confiança.

^a^ Dados ausentes: tipo de PC n = 102 (13,1%); topografia n = 197 (25,4%); nível do GMFCS n = 29 (3,7%); nível do MACS n = 38 (4,9%); comprometimento intelectual n = 111 (14,3%); limitações de fala n = 36 (4,6%); comorbidades n = 18 (2,3%).

^b^ As variáveis incluídas no modelo final estão destacadas em negrito (isto é, aquelas significativas na análise bivariada).

Indivíduos deambuladores com PC (aqueles classificados nos níveis I–III do GMFCS) apresentaram maior frequência escolar em comparação com indivíduos não deambuladores (níveis IV e V do GMFCS). Indivíduos não deambuladores apresentaram *odds ratio* não ajustadas significativamente menores de frequentar a escola (OR não ajustada = 0,36 [0,1–0,7]; p = 0,02) em comparação com indivíduos nos níveis I a III do GMFCS. De forma semelhante, indivíduos com limitações de fala apresentaram menor probabilidade de frequentar a escola em comparação com aqueles sem limitações (OR não ajustada = 0,20 [0,4–0,9]; p = 0,04) (Tabela 3). Outras características clínicas, como níveis do MACS, presença de deficiência intelectual, comprometimentos visuais ou outras comorbidades, não estiveram significativamente associadas à frequência escolar (p > 0,05).r.

### Fatores associados com frequência escolar nos indivíduos com PC

As variáveis que apresentaram associação significativa com a frequência escolar na análise não ajustada foram posteriormente incluídas na análise multivariada. Todas as variáveis incluídas foram consideradas adequadas para o modelo, uma vez que não houve evidência de multicolinearidade (fator de inflação da variância <3).

Idade, nível do GMFCS e comprometimento de fala estiveram significativamente associados à frequência escolar de indivíduos brasileiros com PC no modelo de regressão ajustado (Tabela 4). O aOR para frequentar a escola foi de 0,49 (IC95% = 0,3–0,7; p = 0,001) entre adolescentes e jovens adultos com PC, indicando que esse grupo apresenta maior probabilidade de não frequentar a escola em comparação às crianças. O aOR foi de 0,29 (IC95% = 0,1–0,4; p = 0,001) entre aqueles classificados nos níveis IV e V do GMFCS, indicando que indivíduos não deambuladores têm menor probabilidade de frequentar a escola em comparação aos deambuladores. O aOR foi de 0,12 (IC95% = 0,1–0,3; p = 0,002) entre aqueles com comprometimentos de fala, indicando que indivíduos com limitações de comunicação têm menor probabilidade de frequentar a escola em comparação com aqueles sem essas limitações.

**Tabela 4:** Fatores associados à frequência escolar entre crianças e jovens com paralisia cerebral no RBPC.

Fatores	Odds ratio ajustado (CI95%)	*p*
Idade (anos)		
4–12	Ref.	
13–21	0.49 (0.3–0.7)	0.001
Nível GMFCS		
Deambulador (níveis I–III)	Ref.	
Não deambulador (níveis IV‐V)	0.29 (0.1–0.4)	0.001
Limitações na fala		
Não	Ref.	
Sim	0.12 (0.1–0.3)	0.001

Abreviações: RBPC, Registro Brasileiro de Paralisia Cerebral; IC, intervalo de confiança.

### Interpretação dos resultados e contribuições do grupo PPI

A Figura 2 apresenta os resultados em linguagem simples, conforme elaborado pelo grupo PPI do estudo. Essa figura foi criada para facilitar a tradução do conhecimento entre as partes interessadas e incluir *insights* que possam melhor promover a frequência escolar entre indivíduos com PC. Com base nos resultados apresentados, o grupo PPI se reuniu com os pesquisadores e contribuiu com reflexões importantes que podem explicar os achados. A seção de Discussão é baseada nessas reflexões e no que foi considerado relevante para discussão, de acordo com nossos resultados.

**Figura 2:**
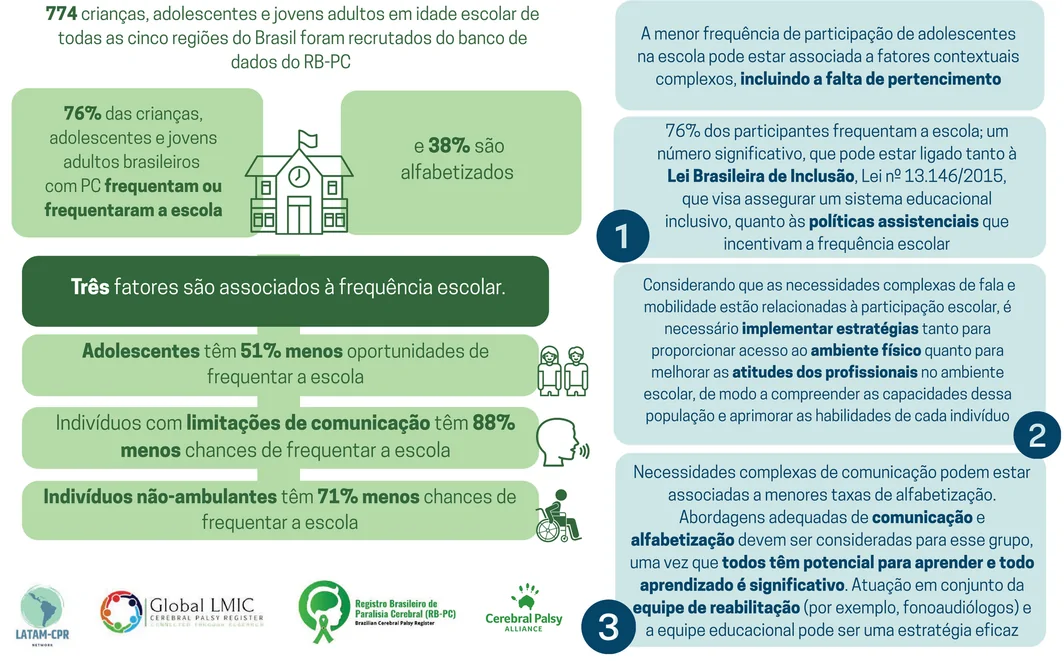
Infográfico dos resultados do estudo.

## DISCUSSÃO

O principal objetivo deste estudo foi determinar fatores associados à frequência escolar entre indivíduos brasileiros com PC. Até onde sabemos, este é o primeiro estudo que avaliou fatores associados à frequência escolar nessa população utilizando dados de base populacional. Observamos que, embora a maioria das pessoas com PC no Brasil frequente a escola, fatores‐chave como idade, nível de função motora e limitações de fala estão fortemente associados à frequência escolar.

De acordo com este estudo, 76% dos indivíduos com PC frequentam a escola — um achado positivo, considerando que as taxas de frequência em outros países de baixa e média renda são muito menores, frequentemente variando entre <12% e 40%.^6^, ^8^, ^9^, ^10^, ^11^ Esses resultados destacam o impacto da Lei Brasileira de Inclusão da Pessoa com Deficiência, da Política Nacional de Educação Especial e do Estatuto da Pessoa com Deficiência (Lei 13.146/2015), que garantem igualdade de oportunidades educacionais, acesso a escolas regulares próximas e proíbem a exclusão ou negligência.^15^, ^25^, ^26^ Essas políticas estão alinhadas com declarações internacionais sobre a escolarização de pessoas com deficiência, como o Manifesto da PC: “*estudantes com paralisia cerebral não devem ser excluídos do sistema educacional regular*”.^27^, ^28^ A implementação bem‐sucedida dessas políticas pode ajudar a explicar as maiores taxas de frequência escolar entre indivíduos com PC no Brasil. No entanto, apesar desse robusto arcabouço legal, lacunas na participação escolar ainda persistem, especialmente entre aqueles com deficiências mais severas.

É importante enfatizar que a frequência (isto é, estar presente) é apenas um componente do conceito mais amplo de participação. A participação em situações da vida envolve a interação de múltiplos fatores. Segundo Imms et al.,^1^ dois construtos principais definem a participação: frequência e envolvimento, sendo o envolvimento a experiência subjetiva de participação durante a presença. Ambos os construtos são influenciados por diversos fatores intrínsecos e extrínsecos. Embora a frequência seja um aspecto necessário e importante da participação escolar, ela não é suficiente para garantir um envolvimento significativo. Embora este estudo não tenha medido diretamente o envolvimento escolar, o achado de que 62% dos indivíduos com PC que frequentam a escola não são alfabetizados pode sugerir envolvimento limitado nas atividades escolares. Essa falta de envolvimento pode, por sua vez, afetar negativamente a continuidade da frequência escolar, uma vez que crianças alfabetizadas têm maior probabilidade de permanecer na escola. Esses resultados reforçam a ideia de que frequência e envolvimento são mutuamente reforçadores e que maiores esforços são necessários para melhorar a qualidade da experiência acadêmica de indivíduos com PC no Brasil.

As limitações de fala estiveram associadas à não frequência escolar em indivíduos com PC. Na população geral, crianças com comprometimentos de fala frequentemente enfrentam desafios na escola, não apenas em termos de desempenho acadêmico, mas também no engajamento geral com a aprendizagem.^29^ O uso de comunicação aumentativa e alternativa pode ser uma estratégia poderosa para apoiar aqueles com limitações de fala mais severas no ambiente escolar. No entanto, diversas barreiras, como acesso inconsistente à comunicação aumentativa e alternativa e a falta de conhecimento e treinamento de professores ou equipe escolar, podem dificultar seu uso eficaz nas escolas brasileiras.^30^ Para prevenir o absenteísmo escolar em estudantes com limitações de fala, é essencial enfrentar essas barreiras, promovendo educação inclusiva e garantindo igualdade de oportunidades de aprendizagem. Uma estratégia possível para reduzir essas barreiras é a participação ativa de profissionais de saúde no ambiente escolar, contribuindo para a inclusão dos estudantes e a capacitação da equipe.

Indivíduos não deambuladores com PC no Brasil apresentam menor probabilidade de frequentar a escola, um padrão também observado em outros países de baixa e média renda, como Bangladesh, Sudão, Índia, Uganda e Benim.^6^, ^8^, ^9^, ^10^, ^11^ Essa menor frequência pode decorrer de maior dependência de terceiros para mobilidade e do acesso limitado a tecnologias assistivas.^6^ Embora dados brasileiros sugiram que as escolas apresentam menos barreiras e mais adaptações do que outros ambientes,^31^ e os participantes deste estudo tenham relatado possuir dispositivos assistivos adequados, essas adaptações ainda podem ser insuficientes para prevenir o absenteísmo. Barreiras relacionadas ao transporte e a limitações financeiras, falta de capacitação da equipe escolar e crenças culturais sobre o potencial educacional de indivíduos não deambuladores podem também limitar a participação escolar. Pesquisas futuras devem investigar o impacto desses fatores na frequência escolar de indivíduos não deambuladores.

Adolescentes e jovens adultos brasileiros com PC frequentam menos a escola do que crianças com PC. Esses achados são consistentes com dados do registro de PC de Bangladesh, que também identificaram esse fator etário como associado à frequência escolar.^6^ Em geral, adolescentes com PC tendem a apresentar menor participação não apenas na escola, mas também em outras áreas da vida.^32^ Diversos fatores podem contribuir para a redução da frequência escolar entre adolescentes brasileiros, incluindo bullying e desafios relacionados ao transporte e à mobilidade, especialmente para indivíduos não deambuladores.^33^, ^34^ Outro ponto importante a considerar é que, de acordo com a legislação brasileira, a frequência escolar é obrigatória apenas até os 17 anos. Após essa idade, adolescentes e jovens adultos com baixo desempenho acadêmico podem sentir‐se menos motivados a continuar os estudos e eventualmente abandonar a escola. São necessários esforços significativos para promover a participação de adolescentes e jovens adultos com PC na escola e em outras áreas da vida.

### Limitações e direções para futuros estudos

Este estudo apresenta algumas limitações que merecem ser destacadas. Primeiramente, devido à estrutura das entrevistas do RBPC, foi possível captar principalmente a frequência escolar com base em relatos de pais e cuidadores, sem informações sobre o envolvimento escolar e a frequência real baseada em registros das escolas. Como os aspectos de envolvimento são fundamentais para compreender a inclusão escolar, estudos futuros devem também explorar os fatores que influenciam o envolvimento na participação em contextos escolares. Em segundo lugar, não foi possível avaliar se os participantes tinham acesso a suporte ou recursos adequados especificamente relacionados à frequência escolar. O acesso à tecnologia assistiva e a profissionais escolares adequadamente capacitados pode desempenhar um papel importante no apoio à participação escolar. Em terceiro lugar, não foi possível avaliar as limitações de fala e comunicação dos indivíduos utilizando instrumentos mais detalhados, como o Sistema de Classificação da Função de Comunicação. Estudos futuros devem investigar como diferentes níveis de habilidade comunicativa entre indivíduos com PC estão associados à frequência escolar. Por fim, as características clínicas puderam ser confirmadas em prontuários de saúde e por clínicos em apenas alguns participantes; a maior parte das informações foi obtida exclusivamente por meio de relatos dos pais.

Os dados do nosso estudo são consistentes com estudos brasileiros recentes de base populacional sobre PC, incluindo a distribuição dos níveis funcionais, características clínicas e socioeconômicas, e a menor frequência escolar observada entre indivíduos classificados em níveis IV e V do GMFCS.^17^, ^35^ Portanto, embora os dados do presente estudo tenham sido coletados entre 2024 e 2025, acreditamos que nossos achados sejam amplamente representativos da população brasileira com PC. No entanto, assim como em outros estudos brasileiros de base populacional, a maioria dos dados foi obtida da região Sudeste, a área mais rica do país.^17^, ^35^ Assim, uma proporção substancial dos participantes pode ter maior acesso a serviços de educação e reabilitação. Dadas as marcantes desigualdades socioeconômicas entre as regiões brasileiras, esses achados podem não refletir plenamente a realidade de áreas menos favorecidas, particularmente na região Norte, onde os dados sobre indivíduos com PC ainda são escassos. À medida que o RBPC se expande geograficamente e o período de coleta de dados se amplia, estudos futuros estarão mais bem posicionados para fornecer uma compreensão mais abrangente da frequência escolar, especialmente em relação a fatores contextuais e socioeconômicos em regiões de baixa renda do Brasil.

De acordo com a legislação brasileira, a frequência escolar é obrigatória apenas até os 17 anos de idade. Como mencionado anteriormente, esse pode ser um dos principais motivos para o absenteísmo escolar nessa faixa etária. Devido à pequena proporção de adolescentes e jovens adultos na amostra, não foi possível analisá‐los como um grupo separado e avaliar com precisão a associação entre essa faixa etária e a frequência escolar. Estudos futuros com amostras maiores de adolescentes e jovens adultos permitirão uma análise mais adequada dessa hipótese.

Embora o estudo apresente poder estatístico adequado para detectar diferenças nas análises, alguns intervalos de confiança permaneceram amplos e vários valores de p ficaram próximos ao limiar de significância; portanto, os resultados devem ser interpretados com cautela. Isso ocorre principalmente porque, apesar do grande tamanho amostral global, várias categorias apresentaram pequeno número de observações, o que reduz a precisão das estimativas. Além disso, a heterogeneidade clínica e sociodemográfica dos participantes aumenta a variabilidade no modelo ajustado, levando a estimativas mais conservadoras. Dessa forma, a amplitude dos intervalos de confiança reflete a distribuição desigual das categorias e a variabilidade intrínseca da amostra, e não necessariamente a ausência de uma associação verdadeira. Ademais, análises futuras, incluindo modelagem multinível com agrupamento por região, podem ajudar a esclarecer influências socioeconômicas contextuais que não foram detectadas neste modelo inicial. Isso é particularmente relevante no Brasil, onde os indicadores socioeconômicos apresentam forte variação entre as regiões.

### Conclusões

As taxas de frequência escolar são mais altas no Brasil do que em outros países de baixa e média renda. No entanto, idade, nível do GMFCS e limitações de fala estão associados à não frequência escolar. O fortalecimento do suporte à comunicação e da acessibilidade é essencial para melhorar a frequência escolar.

#### RECONHECIMENTOS

Agradecemos a todos os pesquisadores, colaboradores, estudantes, membros do grupo EPP e profissionais que fazem parte do RBPC. Agradecemos à Universidade Federal de Minas Gerais e ao Conselho Nacional de Desenvolvimento Científico e Tecnológico (CNPq) pelo apoio institucional. Este estudo foi financiado pela Coordenação de Aperfeiçoamento de Pessoal de Nível Superior (CAPES), por meio de bolsas de doutorado e de pesquisa. Tambemagradecemos a Cerebral Palsy Alliance Research Foundation pelofinanciamentoinicialpormeio do projeoto Global Low and Middle‐Income Cerebral Palsy Registers (numero deconcessao PRG00125). Como o inglês não é a primeira língua da maioria dos autores, reconhecemos o uso do ChatGPT (OpenAI, 2025) para auxiliar na melhoria da clareza, gramática e fluência da redação da versão em inglês deste estudo. Todo o conteúdo foi revisado e verificado pelos autores para garantir sua precisão e integridade. The Article Processing Charge for the publication of this research was funded by the Coordenação de Aperfeiçoamento de Pessoal de Nível Superior ‐ Brasil (CAPES) (ROR identifier: 00x0ma614).Osautoresdeclaramquenaoexisteconflitosdeinteresse. Os dados que sustentam os achados deste estudo estao disponiveis com o autor correspondente, mediante a solicitaçao.

## INFORMAÇÃO de FOMENTO

Este estudo foi financiado pela Coordenação de Aperfeiçoamento de Pessoal de Nível Superior (CAPES), por meio de bolsas de doutorado e de pesquisa.

## DECLARAÇÃO de CONFLITOS de INTERESSE

Os autores declaram não ter conflitos de interesse.

## Supporting information


**Apêndice S1**: Formulário do Registro Brasileiro de Paralisia Cerebral (RBPC)

## Data Availability

Os dados que sustentam as conclusões deste estudo não estão disponíveis publicamente devido a restrições de privacidade e éticas.
